# Efficacy of 18-fluoro deoxy glucose-positron emission tomography computed tomography for the detection of colonic neoplasia proximal to obstructing colorectal cancer

**DOI:** 10.1097/MD.0000000000011655

**Published:** 2018-08-03

**Authors:** Daisuke Hojo, Toshiaki Tanaka, Miwako Takahashi, Koji Murono, Shigenobu Emoto, Manabu Kaneko, Kazuhito Sasaki, Kensuke Otani, Takeshi Nishikawa, Keisuke Hata, Kazushige Kawai, Toshimitsu Momose, Hiroaki Nozawa

**Affiliations:** a Department of Surgical Oncology; b Division of Nuclear medicine, Department of Radiology, Faculty of Medicine, University of Tokyo; c Department of Radiology, International University of Health and Welfare, School of Medicine, Japan.

**Keywords:** aspect ratio, obstructing colorectal cancer, positron emission tomography, sensitivity, standard uptake value max

## Abstract

Identification of secondary colonic neoplasia proximal to obstructing colorectal cancer is essential for determining the range of colorectal resection.

We examined the accuracy of 18-fluoro deoxy glucose-positron emission tomography (FDG-PET) for detection of colonic neoplasia.

We recruited patients with obstructing colorectal cancer from our registry. Preoperative FDG-PET was performed, and the detection rate for colonic neoplasia was estimated. Preoperative colonoscopy or postoperative colonoscopy within a year after operation was employed as the indexed standard.

Ninety-three patients were included in this study. Colonic neoplasia proximal to obstruction was confirmed in 83 cases. The sensitivity and positive predictive value of FDG-PET were 25.3% and 77.8%, respectively. The sensitivity was higher in larger lesions (3.2% for <5 mm, 29.4% for 6–10 mm, 45.5% for 11–20 mm, and 71.4% for >21 mm) and in higher pathological grade lesions (14.6% for low-grade adenoma, 38.5% for high-grade adenoma, 66.7% for carcinoma in situ, and 100% for invasive carcinoma). The round shape in PET images was a predictor for neoplasia, with an area under the curve of 0.75293 at an aspect ratio of 1.70.

FDG-PET should be used as a screening modality for invasive colorectal cancer (CRC) proximal to obstructing colorectal cancer.

## 1 Introduction

Colorectal cancer (CRC) is one of the most common worldwide cancers. It accounts for 14.5% of all malignancies detected in Japan and is a major cause of death. It is reported that the multiple synchronous CRC occurs in 4.8% to 12.4% of all CRC cases^[1,2]^; therefore, it is essential to examine through the pan-colon to confirm the location of all lesions before deciding the form of colorectal resection.^[3]^

Obstructing CRC accounts for 7% to 14% of all CRC cases at the time of diagnosis.^[4–7]^ Although colonoscopy provides high sensitivity and specificity for the detection of colorectal lesions,^[8]^ it is not able to search proximal to the obstruction because the CRC occupying the lumen interrupts passage of the scope.^[9]^ When we determine the range of colorectal resection, detection, and inclusion of proximal neoplasia is crucial for avoiding a second operation. Alternative options for examining the proximal colon are CT colonography (CTC)^[10,11]^ and enema x-ray with water-soluble contrast media,^[12,13]^ if the stenosis is not severe. Intraoperative endoscopy^[3,14]^ or colonoscopy after placement of a colonic self-expanding metallic stent (SEMS)^[9,15]^ is applied if the stenosis is severe.

However, these modalities have some disadvantages, such as their technical difficulty and risks to the patient.^[9,10,14,15]^ In contrast, 18-fluorodeoxyglucose positron emission tomography (FDG-PET) provides a non-invasive option for the investigation of obstructing CRC, regardless of the stenotic condition.^[16,17]^ CTC requires adequate bowel preparation^[18]^ and carries the risk of nausea with gas insufflation to the intestine^[19]^; furthermore, radiologists need technical training to achieve high sensitivity for colonic lesions when interpreting CTC images.^[20]^ Colonic stent placement prior to preoperative colonoscopy and enema has the risk of perforation.^[21,22]^ Although it has been shown that intraoperative colonoscopy proximal to the obstruction does not affect the postoperative course, this procedure takes additional operation time; in addition, it cannot be used with laparoscopic surgery, an approach to colorectal surgery that is becoming increasingly common because of its less invasive nature.^[3]^ FDG-PET is a less invasive modality for detecting colonic lesions proximal to the obstruction, regardless of the operative approach.

FDG-PET is a useful modality for detecting not only CRC but also adenoma in the large intestine. Application of FDG-PET for the surveillance of colorectal lesions was attempted in previous studies^[18,23–26]^; however, there are only a few studies reporting the efficacy of FDG-PET for obstructing CRC.^[16,17]^ In these limited studies, they argue that the high sensitivity of FDG-PET would permit the exclusion of proximal colorectal lesion although the sensitivity is affected when the lesion is small or less invasive.^[27–30]^ We consider it is necessary to accumulate data and establish diagnostic criteria to make FDG-PET practically useful.

In this study, we examined the accuracy of FDG-PET in identifying proximal synchronous lesion for cases of obstructing CRC. In addition, SUV max^[16,31,32]^ was analyzed in association with the size and pathological grade of the colorectal lesions. This study reported the features of FDG-PET, suggesting the possibility of colorectal lesions proximal to obstructing CRC, with the expectation that this application could be clinically useful, especially when the range of resection for CRC cancer must be determined.

## 2 Material and method

### 2.1 Study design

This was a retrospective study in patients with obstructing CRC who underwent surgical resection following FDG-PET at Tokyo University Hospital. The findings of FDG-PET in the colon proximal to the obstruction were compared with reference data, which were collected during colonoscopy or from the resected colonic specimen. The research was approved by the ethics committee at Tokyo University Hospital.

### 2.2 Patients

We recruited patients from our registry who underwent colorectal resection from April 2007 to March 2016. Those who underwent ileocecal resection, right hemicolectomy, or extended right hemicolectomy were excluded because lesions proximal to the obstruction would be included in the resected specimen and would not influence the type of resection. Patients with inflammatory bowel disease were also excluded to avoid the influence on FDG-accumulation.^[33,34]^ Because we referred to perioperative colonoscopy as a standard index for proximal lesions, those who failed to undergo colonoscopy before and within 1 year after operation were excluded from the analysis.

### 2.3 FDG-PET

FDG-PET was performed as a routine preoperative examination for obstructing CRC. The patients kept fasting 5 hours before the examination and had blood sugar level under 150 mg/dL. Oral medication for diabetes mellitus and insulin injections were discontinued. Patients were injected with 4.5 MBq/kg (0.12 mCi/kg) of 18-fluodeoxy glucose (18-FDG), remained resting on the bed for 30 minutes, and FDG-PET scan started 50 minutes after injection using a PET/CT scanner (Aquiduo, Toshiba Medical System, Otawara, Japan). These images were synthesised with those from computed tomography (CT) to facilitate the interpretation.

An expert nuclear medicine physician re-read these images blindly at first time, detected the abnormal accumulation in the proximal colon and then, recorded its location, number of lesions, and SUV max. Long and short axes of the area with SUV >2.5 were measured to calculate the aspect ratio for further analysis.

### 2.4 Reference index of the proximal colon to the obstruction

Findings from colonoscopy proximal to the obstruction were employed as reference standards. Colonoscopy was performed as a preoperative screening following the placement of a SEMS for obstructing CRC, as an intraoperative colonic endoscopy or as a postoperative colonoscopy within a year after operation. All procedures were performed by expert gastroenterologists.

Proximal polyps >5 mm or indicative of adenoma or carcinoma as judged by its surface structures were resected or biopsied for pathological confirmation. The features of neoplasia, including location, size, morphological classification according to the “Paris endoscopic classification of superficial neoplastic lesions in the digestive tract” and pathology were recorded. In addition, any colorectal lesion detected in the resected specimen was also used for reference.

### 2.5 Statistical analysis

All findings by radiologist from FDG-PET images were compared with those of colonoscopy. The sensitivity of neoplasia per lesion detected by radiologists from FDG-PET images was calculated. The sensitivities stratified by size, pathological grade, and morphological type were also calculated. The relationship between size, tumor malignancy, and SUV max was determined using Spearman analysis. The aspect ratio in the area whose SUV max was >2.5^[32,35]^ was analyzed in relation to the existence of colonic neoplasia by Wilcoxon test and receiver operating characteristic (ROC) curve. The analyses were 2-sided and statistical significance was defined as a *P*-value <.05. These statistical calculations were performed by JMP Pro 12.2.0 for Mac (SAS Institute Inc. Cary, NC)

## 3 Results

### 3.1 Patients and neoplasia characteristics

Patient characteristics are reported in Table 1. All patients underwent colonoscopy, without morbidity. There was no operative mortality for the primary CRC. There were 60 men and 33 women, and mean age was 66 years (37–86 years). The number of cases for each location of the primary obstructing CRC was 6 in the transverse colon, 9 in the descending colon, 45 in the sigmoid colon, and 33 in the rectum. No patients had synchronous obstructing cancer. The tumor node metastasis/union for international cancer control (TNM/UICC) staging was as follows; Stage I in 2 cases, Stage II in 34 cases, Stage III in 31 cases, and Stage IV in 26 cases. Obstruction of 2 cases in Stage I was caused for their large size. Open surgery was performed in 36 patients, and laparoscopic surgery was performed in 57 patients, of whom robot-assisted surgery was performed in 2. A SEMS was placed for 9 obstructing CRC as a bridge to surgery. Seven of them had preoperative colonoscopy via the SEMS. Fourteen patients underwent intraoperative colonoscopy and 84 patients underwent colonoscopy within a year after operation. Twelve patients underwent total colonoscopy >2 times preoperatively. We found 83 colorectal neoplasia proximal to obstruction.

**Table 1:**
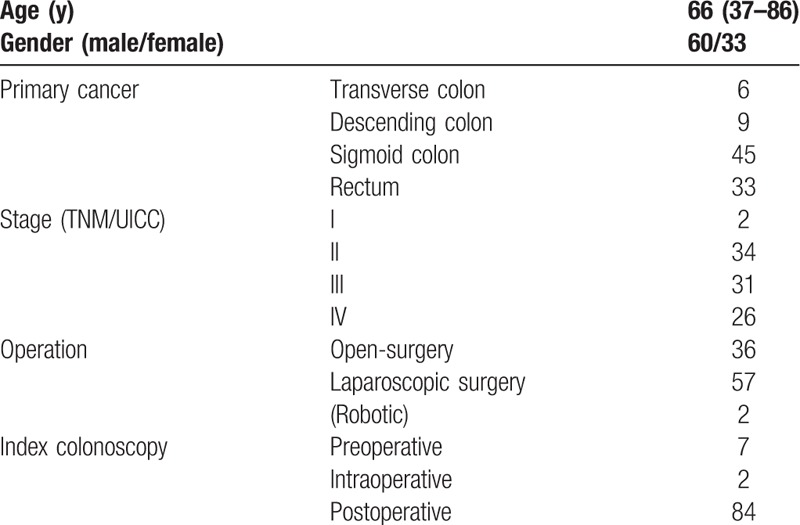
Patients characteristics.

### 3.2 Sensitivity for colonic neoplasia determined by FDG-PET

In all 83 lesions, 21 were positive in FDG-PET by nuclear medicine physician and 62 were not. The overall sensitivity was 25.3% (21/83) and the positive predictive value was 77.8% (21/27) (Table 2).

**Table 2:**
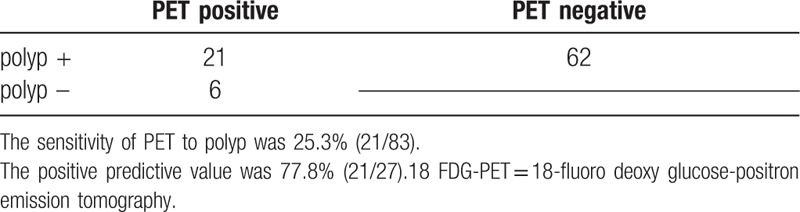
Sensitivity and positive predictive value of 18 FDG-PET (per lesion).

The sensitivities stratified by size, pathology, or morphology are shown in Table 3. The sensitivity was significantly higher for larger tumor sizes; 1 to 5 mm: 3.2% (1/31), 6 to 10 mm: 29.4% (10/34), 11 to 20 mm: 45.5% (5/11), and 21 mm: 71.4% (5/7), with a *P*-value <.001. The sensitivity stratified by pathology revealed higher for higher pathological grade tumors; hyper-plastic polyps: 0% (0/2), adenoma with low-grade dysplasia: 14.6% (7/48), high-grade dysplasia: 38.5% (5/13), mucosal cancer: 66.7% (2/3), and invasive cancer: 100.0% (5/5), with a *P*-value <.001. Furthermore, there was a significant difference in sensitivity with respect to morphology; flat type: 38.4% (5/13), protrude type: 16.9% (11/65), type 1: 100% (1/1), and type 2: 100%(4/4); however, we consider this difference was only the result of type 1 and type 2 tumors consisting of invasive cancer that were marked high sensitivity. In all 93 patients, 22 were positive by nuclear medicine physician and 71 were not. And overall sensitivity was 46.1% (18/39) and the positive predictive value was 81.8% (18/22) (Table 4).

**Table 3:**
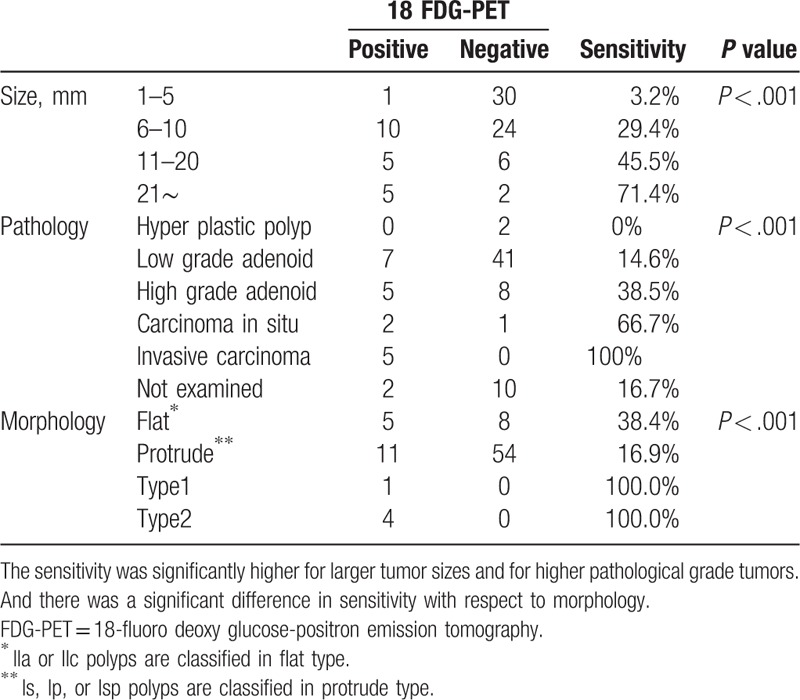
Sensitivity and positive predictive value of 18 FDG-PET.

**Table 4:**
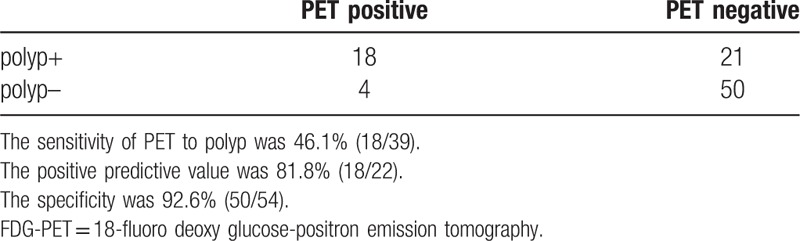
Sensitivity and positive predictive value of 18 FDG-PET (per patient).

### 3.3 Axis ratio of 18FDG-accumulations

**Figure 1:**
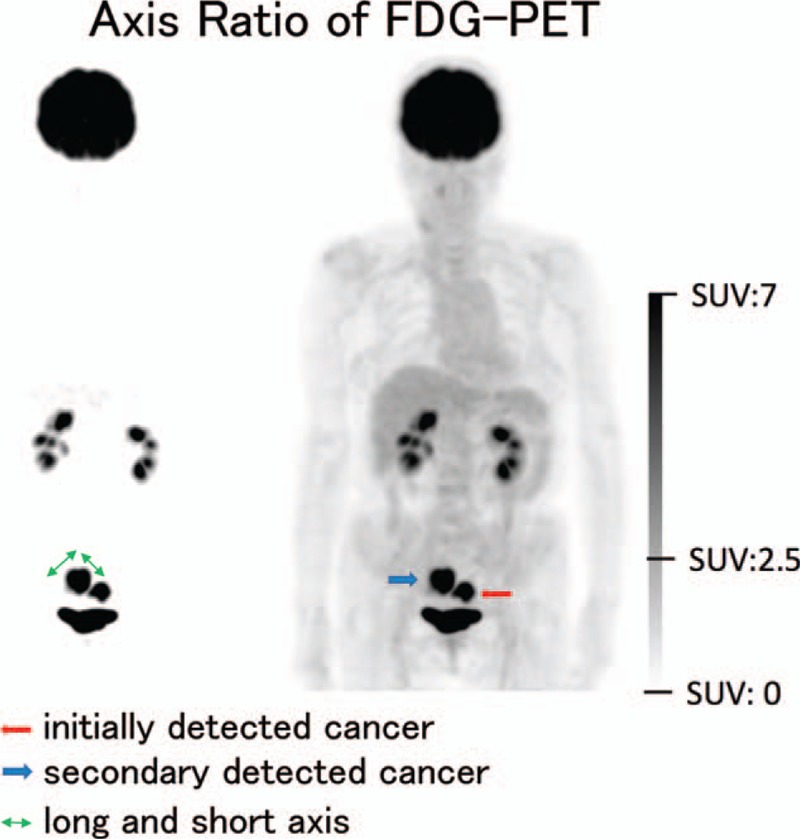
An example of 18FDG-accumulating area with SUV max ≥2.5; the aspect ratio (longitude to latitude) was calculated and. In fact, this case was invasive CRC. CRC = colorectal cancer, 18FDG = 18-fluoro deoxy glucose, SUV = standard uptake value.

**Figure 2:**
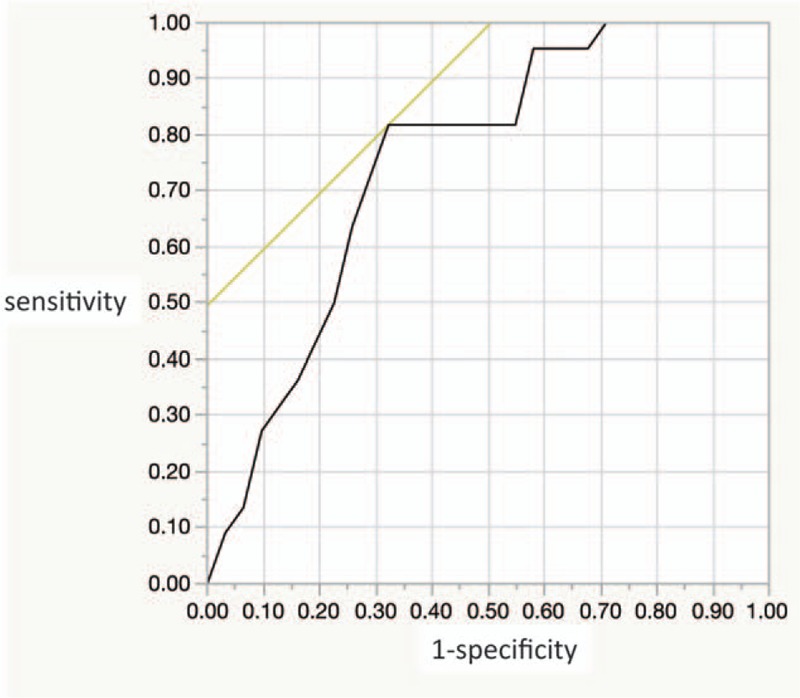
ROC curve of the aspect ratio on PET images predicting the proximal colonic neoplasia. The AUC was 0.75293 when the cut-off value was 1.70. AUC = area under the curve, PET = positron emission tomography, ROC = receiver operating characteristic.

There were 60 locations with SUV max ≥2.5, which was the cut-off value often used in previous reports.^[32,35]^ Twenty-five adenoma and cancer were detected in these locations and 35 FDG-accumulation did not reflect colonic polyps. (An example is shown in Fig. 1.) Then, we determined if the aspect ratio in the accumulated area was associated with the existence of adenoma or cancer. From ROC curves, we showed that a cut-off value of 1.70 for the aspect ratio marked the highest detectable accuracy for colonic polyps with an area under curve (AUC) of 0.75293 (Fig. 2). If SUV max ≥2.5 and aspect ratio <1.70 were used as criteria, the sensitivity was 25.3% (21/83) and the positive predictive value was 67.7% (25/31).

### 3.4 Synchronous invasive CRC cases

Five invasive CRCs were detected by FDG-PET and confirmed intraoperatively as an indurative or a visually apparent mass (Table 5). Four of the synchronous cancers were resected at the time of the primary operation to avoid the need for a second operation. Treatment for the fifth synchronous cancer was delayed because of a heart complication during the operation. However, colonoscopy was performed soon after the operation and the patient then underwent an additional operation. The presence of synchronous CRC was not shown clearly on CT, which meant that radiologists only found one of these CRCs (Case no. 5).

**Table 5:**
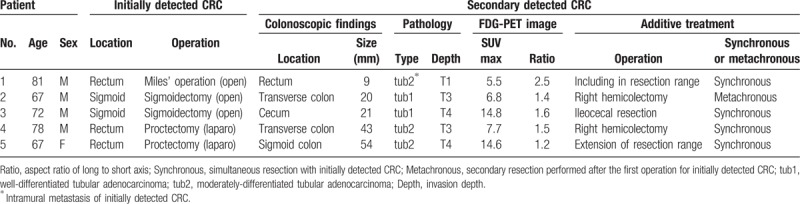
Synchronous invasive CRC cases.

## 4 Discussion

In this study, we examined the ability to detect proximal neoplasia in obstructing CRC by FDG-PET and showed that the sensitivity of polyps by FDG-PET was elevated with the polyps larger size and higher pathological grade. We also found that a round accumulation was another diagnostic observation indicative of colonic neoplasia. Furthermore, all 5 invasive CRCs, which require colorectal resection, were detected by FDG-PET preoperatively as previous reports suggested.^[16,17]^ This result suggests that FDG-PET is an essential preoperative diagnostic modality for avoiding overlooking colonic lesions, which otherwise should be resected by a second operation. FDG-PET plays an essential role in treating obstructing CRC.

Although some smaller and lower pathological grade polyps were detected by FGD-PET, the sensitivity was not similar to that in previous reports.^[27]^ The total sensitivity for adenoma was 19.0% (12/63) in this study for many small adenomas contained in our study. This result indicates that, even when FDG-PET of the proximal colon is negative, postoperative colonoscopy should still be performed to remove small adenomas, which can grow to become cancerous.^[36]^

As we have shown the SUV max of the adenoma was significantly lower than cancer. The cut-off value of SUV max was supported by ROC analysis. Na et al^[32]^ proposed SUV max of 5.8 as a cut-off value, with the maximum sum of sensitivity and specificity; however, the sensitivity was only 71.8% even for high-grade dysplasia and carcinoma. Sekiguchi et al^[25]^ proposed a cut-off value for SUV max of 6.3 for advanced neoplasia; however, the sensitivity was only 52.4% even for advanced neoplasia. (In our study, the sensitivity of advanced neoplasia was 43.3% [13/30].) These reports support that evaluation of only SUV max did not provide satisfactory sensitivity for the screening of colorectal malignancies. Therefore, we consider that other indicators are needed for the screening with a focus on shape.

Focal round uptake was often interpreted as neoplasia, while a diffuse pattern or snake-like longitudinal uptake was considered as physical activation like intestinal peristalsis or colitis.^[25,37,38]^ Malignant lesions derived from other primary tumors (or metastasis) and hyper-plastic polyps are also imaged as focal accumulation on FDG-PET.^[32,37]^ Although these morphological features were taken into consideration when nuclear medicine physician interpreted the images of FDG-PET, there was no report qualifying the focal shape for the accumulation using a subjective method. Therefore, we conducted this study and proposed that an aspect ratio of 1.70 was a candidate as a predictor of colonic neoplasia; however, the sensitivity was low. Therefore, we consider that although FDG-PET is a less invasive screening modality it should be combined with other screening modalities.

For clinical application, CTC and enema are known as useful modalities. Concerning the accuracy of CTC for obstructing CRC, Park et al^[10]^ reported sensitivities for non-advanced adenoma (≥6 mm), advanced adenoma and adenocarcinoma of 65.8% (48/73), 77.2% (44/57), and 100% (9/9), respectively, and proposed lesions >15 mm on CTC images should be a criterion for CRC suspicion. Although CTC had a sensitivity comparable to FDG-PET, the crucial limitation was that it is not applicable for complete obstruction because CTC requires an adequate bowel.^[11]^ Double-contrasted gastrografin enema, another representative modality, also has this limitation,^[12]^ and a lower detection rate.^[18]^ Despite these limitations, we consider that to compensate for the low sensitivity of FDG-PET, CTC or gastrografin enema should be employed in clinical use if possible. As another combined method to increase accuracy, a dual time point technique of FDG-PET is used at some institutions to confirm pathological uptake in a questionable lesion.^[16]^

Intraoperative colonoscopy^[3,14]^ or preoperative colonoscopy after placement of a stent^[9,15]^ can be performed even in cases of severely stenotic obstructing CRC. This is the most sensitive and specific modality. As we have reported, detection rate of intraoperative colonoscopy for polyps >9 mm was 91.7% (11/12).^[14]^ To compensate for the accuracy of FDG-PET, additional examination by pre- or intra-colonoscopy are expected to yield high efficacy. Of course, SEMS placement is accompanied by risk of perforation; however, the incident rate is low.^[9,15]^

Screening of the colon proximal to the primary obstruction is necessary for all patients, with the exception of those whose proximal colon is removed during the operative procedure, such as patients undergoing ileocecal resection, right hemicolectomy, or extended right hemicolectomy.^[3]^ We believe that FDG-PET should be used when the sensitivity of other modalities for detecting a colonic lesion is affected by the patients’ condition. For instance, it is difficult to visualize a colonic lesion by CTC or gastrografin enema when it is in the middle of a dense diverticulosis.^[39]^ Stent placement, followed by total colonoscopy is a promising modality for detecting proximal lesions; however, it can sometimes be difficult to perform stenting in the descending colon or splenic flexure,^[40]^ or when obstruction is accompanied by extraluminal fistulae. In such cases, FDG-PET is a good modality to use for colonic screening. Conversely, it would not be appropriate to use FDG-PET for a patient with obstructing colitis because of difficulty in discriminating the colonic lesion from the high FDG accumulation caused by the colitis.^[38]^

There are some limitations for this study. First, this was a retrospective study and the nuclear medicine physician was not blinded in several cases to the information of the existence of neoplasms in proximal colon. Second, the interval between operation and the initial postoperative colonoscopy was not uniform. Third, the sample size was small.

The combination of FDG-PET and other modalities provides a useful approach for obstructing CRC in clinical practice. If FDG-PET suggests the presence of a lesion, colonic stent placement followed by colonoscopy should be performed. Thereafter, colonic lesions can be removed endoscopically if adenomas or carcinomas are detected in situ in the proximal colon preoperatively. If invasive CRC is revealed by preoperative total colonoscopy, the range of colonic resection can be replanned in advance.

## Author contributions

**Conceptualization:** Daisuke Hojo, Toshiaki Tanaka.

**Data curation:** Daisuke Hojo.

**Supervision:** Toshiaki Tanaka, Miwako Tkahashi, Hiroaki Nozawa, Kazushige Kawai, Toshimitsu Momose, Koji Murono, Shigenobu Emoto, Manabu Kaneko, Kazuhito Sasaki, Kensuke Otani, Takeshi Nishikawa, Keisuke Hata.

**Writing – original draft:** Daisuke Hojo.
